# Diagnostic value of zonulin, citrulline, and novel hematological indices in pediatric celiac disease

**DOI:** 10.1590/1806-9282.20260668

**Published:** 2026-10-30

**Authors:** Melek Bilgin, Hatice Köse, Recai Aci

**Affiliations:** 1 Samsun University, Samsun City Hospital, Department of Medical Microbiology – Samsun, Türkiye.; 2 Samsun City Hospital, Department of Infectious Diseases and Clinical Microbiology – Samsun, Türkiye.; 3 Aydın Adnan Menderes University, Söke Vocational School of Health Services – Aydın, Türkiye.

**Keywords:** Celiac disease, Citrulline, Intestinal barrier function, Inflammation, Biomarkers

## Abstract

**OBJECTIVE:**

The aim of this study was to investigate whether serum zonulin, citrulline, and hemogram-derived inflammatory indices—the systemic immune-inflammation index, systemic inflammation response index, and pan-immune-inflammation value—are associated with newly diagnosed pediatric celiac disease and to characterize their ability to distinguish the study groups.

**METHODS:**

A total of 82 children, comprising 48 patients with celiac disease and 34 healthy controls, were included. Serum zonulin, citrulline, and tissue transglutaminase immunoglobulin A levels and the systemic immune-inflammation index, systemic inflammation response index, and pan-immune-inflammation value were compared between groups. Receiver operating characteristic analysis was used to describe group discrimination within the selected sample, highlighting the limited standalone utility of these markers.

**RESULTS:**

Zonulin, tissue transglutaminase immunoglobulin A, the systemic immune-inflammation index, the systemic inflammation response index, and pan-immune-inflammation value were higher in the celiac group (p≤0.024), whereas citrulline did not differ between groups (p=0.982). The area under the receiver operating characteristic curves of pan-immune-inflammation value (0.644), monocyte count (0.642), the systemic immune-inflammation index (0.614), and the systemic inflammation response index (0.612) indicated poor discrimination and did not support their use as stand-alone diagnostic tests. The systemic inflammation response index and pan-immune-inflammation value were strongly correlated (ρ=0.91, p<0.001); this association should be interpreted in view of their shared blood-count components.

**CONCLUSION:**

Zonulin and hematological inflammation indices are potential markers associated with increased intestinal permeability and systemic inflammation in pediatric celiac disease. Together with tissue transglutaminase immunoglobulin A, these parameters may have an adjunctive role in reflecting disease-related pathophysiological processes; however, their low discriminatory performance precludes their use as stand-alone diagnostic tests.

## INTRODUCTION

Celiac disease is a chronic autoimmune enteropathy triggered by dietary gluten in genetically susceptible individuals. It affects approximately 1% of the global population and can present at any age, with a higher prevalence among females^1,2^. Gluten is the principal environmental trigger, and lifelong adherence to a gluten-free diet remains the only established treatment^3^. Anti-tissue transglutaminase IgA (tTG-IgA) is a key serological marker, and genetic susceptibility is primarily conferred by the HLA-DQ2 and HLA-DQ8 alleles^4,5^. Although the small intestine is the main site of involvement, celiac disease has a broad spectrum of gastrointestinal and extraintestinal manifestations^6^.

Zonulin is a physiological regulator of intestinal tight junctions^7^. In celiac disease, gliadin-induced zonulin release increases paracellular permeability, allowing gluten peptides to cross the epithelial barrier and reach the lamina propria, where the adaptive immune response is initiated^8,9^. Candidate blood- and enterocyte-derived biomarkers have also been investigated in celiac disease^10,11^. Elevated zonulin concentrations may reflect altered intestinal barrier function, although results can vary according to the biological matrix and analytical method used^12^.

Citrulline is a nonessential amino acid produced predominantly by small-intestinal enterocytes and released into the circulation^8,9^. Its plasma concentration has been studied as an indicator of enterocyte functional mass. Nutritional status and intestinal permeability can further influence biomarker interpretation^13^. Because histopathological severity can influence citrulline concentrations, interpretation in celiac disease requires correlation with standardized biopsy findings^10,14^.

Hemogram-derived inflammatory indices, including the neutrophil-to-lymphocyte ratio, platelet-to-lymphocyte ratio, systemic immune-inflammation index (SII), systemic inflammation response index (SIRI), and pan-immune-inflammation value (PIV), have attracted interest as accessible measures of systemic inflammation in celiac disease^15-18^. This cross-sectional pilot study evaluated the associations of serum zonulin, citrulline, and the SII, SIRI, and PIV with newly diagnosed pediatric celiac disease and described their ability to distinguish the selected celiac and control groups.

## METHODS

### Study design and patients

This study was conducted at Samsun University Training and Research Hospital between August 1, 2025, and December 31, 2025, following ethics committee approval. Forty-eight patients aged 1–17 years who were diagnosed with celiac disease in accordance with the European Society for Paediatric Gastroenterology, Hepatology and Nutrition (ESPGHAN) guidelines and were positive for anti-tTG IgA and anti-endomysial antibodies (EMAs) were enrolled. The control group comprised 34 children referred for routine health examinations during the study period who tested negative for anti-tTG IgA and EMA. Patients with chronic conditions other than celiac disease, including type 1 diabetes, autoimmune thyroiditis, autoimmune hepatitis, and Addison disease, were excluded.

### Biochemical analyses

Neutrophil, monocyte, lymphocyte, and platelet counts were determined from complete blood counts obtained using a Sysmex XN-1000 analyser (Sysmex Corporation, Kobe, Japan). Serum aliquots were placed in Eppendorf tubes and stored at −80°C until zonulin and citrulline measurements were performed.

### Biological analyses

Anti-EMAs were detected by indirect immunofluorescence using primate liver sections as the substrate at a screening dilution of 1:10 (Euroimmun AG, Lübeck, Germany). Anti-tTG IgA concentrations were measured with an automated enzyme-linked immunosorbent assay system (Alegria^®^, Orgentec Diagnostika, Germany); results >10 U/mL were interpreted as positive.

Serum zonulin and citrulline concentrations were quantified using commercial enzyme-linked immunosorbent assay kits (BT LAB Bioassay Technology Laboratory, Zhejiang, China). Optical density was measured at 450 nm with a Tecan Infinite M200 PRO microplate reader (Tecan, Austria), and analyte concentrations were calculated from standard curves constructed using recombinant standards of known concentration. The assay range was 0.3–90 ng/mL for zonulin (sensitivity, 0.13 ng/mL) and 0.5–100 ng/mL for citrulline (sensitivity, 0.23 ng/mL).

Ethical approval was obtained from the Non-Interventional Clinical Research Ethics Committee of Samsun University (June 25, 2025; approval no. GOKAEK 2025/13/12).

## RESULTS

The study included 82 children: 48 with celiac disease and 34 healthy controls. The celiac group included 33 females (68.8%) and 15 males (31.2%), whereas the control group included 24 females (70.6%) and 10 males (29.4%). Sex distribution did not differ between groups (χ^2^=0.002, p=0.964; Table 1).

**Table 1:** Sex distribution by disease status.

Group	Female	Male	Total	χ^2^	p-value
Celiac	33 (68.8%)	15 (31.2%)	48	0.002	0.964
Control	24 (70.6%)	10 (29.4%)	34		

Serum zonulin was higher in the celiac group than in the control group (28.75±9.16 vs. 22.96±10.19 ng/mL; p=0.024). Citrulline concentrations did not differ between groups (31.76±9.85 vs. 33.35±9.90 μmol/L; p=0.982). As required by the group definitions, tTG-IgA concentrations were higher in the celiac group (55.46±33.01 vs. 2.76±1.67 U/mL; p<0.001).

The neutrophil count was higher in the celiac group than in controls (4.84±1.94 vs. 3.99±1.45 ×10^3^/μL; p=0.008), as was the monocyte count (0.73±0.20 vs. 0.57±0.16 ×10^3^/μL; p<0.001). Lymphocyte and platelet counts did not differ significantly (p=0.844 and p=0.142, respectively).

The SII (504.72±311.51 vs. 328.28±183.30; p=0.001), SIRI (1.32±0.85 vs. 0.81±0.47; p<0.001), and PIV (263.93±166.22 vs. 150.59±91.44; p<0.001) were higher in the celiac group (Table 2).

**Table 2:** Comparison of biochemical and hematological parameters between the celiac and control groups.

Biomarker	Celiac, mean±SD	Control, mean±SD	p-value
Zonulin (ng/mL)	28.75±9.16	22.96±10.19	0.024
Citrulline (μmol/L)	31.76±9.85	33.35±9.90	0.982
tTG-IgA (U/mL)	55.46±33.01	2.76±1.67	<0.001
Neutrophils (×10^3^/μL)	4.84±1.94	3.99±1.45	0.008
Lymphocytes (×10^3^/μL)	2.94±0.93	2.87±0.79	0.844
Monocytes (×10^3^/μL)	0.73±0.20	0.57±0.16	<0.001
Platelets (×10^3^/μL)	313.11±76.34	291.64±63.47	0.142
SII	504.72±311.51	328.28±183.30	0.001
SIRI	1.32±0.85	0.81±0.47	<0.001
PIV	263.93±166.22	150.59±91.44	<0.001

PIV: pan-immune-inflammation value; SII: systemic immune-inflammation index; SIRI: systemic inflammation response index; tTG-IgA: tissue transglutaminase immunoglobulin A; SD: standard deviation.

The SIRI and PIV were strongly correlated (ρ=0.91, p<0.001). The SIRI correlated with neutrophil count (ρ=0.82, p<0.001), and the SII correlated with neutrophil count (ρ=0.78, p<0.001), PIV (ρ=0.73, p<0.001), and the SIRI (ρ=0.70, p<0.001). Lymphocyte count correlated inversely with the SIRI (ρ=-0.60, p<0.001), PIV (ρ=-0.56, p<0.001), and the SII (ρ=-0.51, p<0.001; Table 3). Because these indices are calculated from overlapping blood-count components, these correlations do not establish independent clinical associations.

**Table 3:** Key positive and negative associations according to Spearman rank correlation analysis.

Variables	ρ (Spearman)	p-value
SIRI and PIV	0.91	<0.001
Neutrophils and SIRI	0.82	<0.001
Neutrophils and SII	0.78	<0.001
SII and PIV	0.73	<0.001
SII and SIRI	0.70	<0.001
Lymphocytes and SIRI	-0.60	<0.001
Lymphocytes and PIV	-0.56	<0.001
Lymphocytes and SII	-0.51	<0.001

PIV: pan-immune-inflammation value; SII: systemic immune-inflammation index; SIRI: systemic inflammation response index

Within the selected sample, receiver operating characteristic analysis yielded area under the receiver operating characteristic curve (AUC) values of 0.644 for PIV, 0.642 for monocyte count, 0.614 for the SII, and 0.612 for the SIRI (Figure 1). These values represent poor group discrimination and are insufficient for stand-alone diagnostic use.

**Figure 1:**
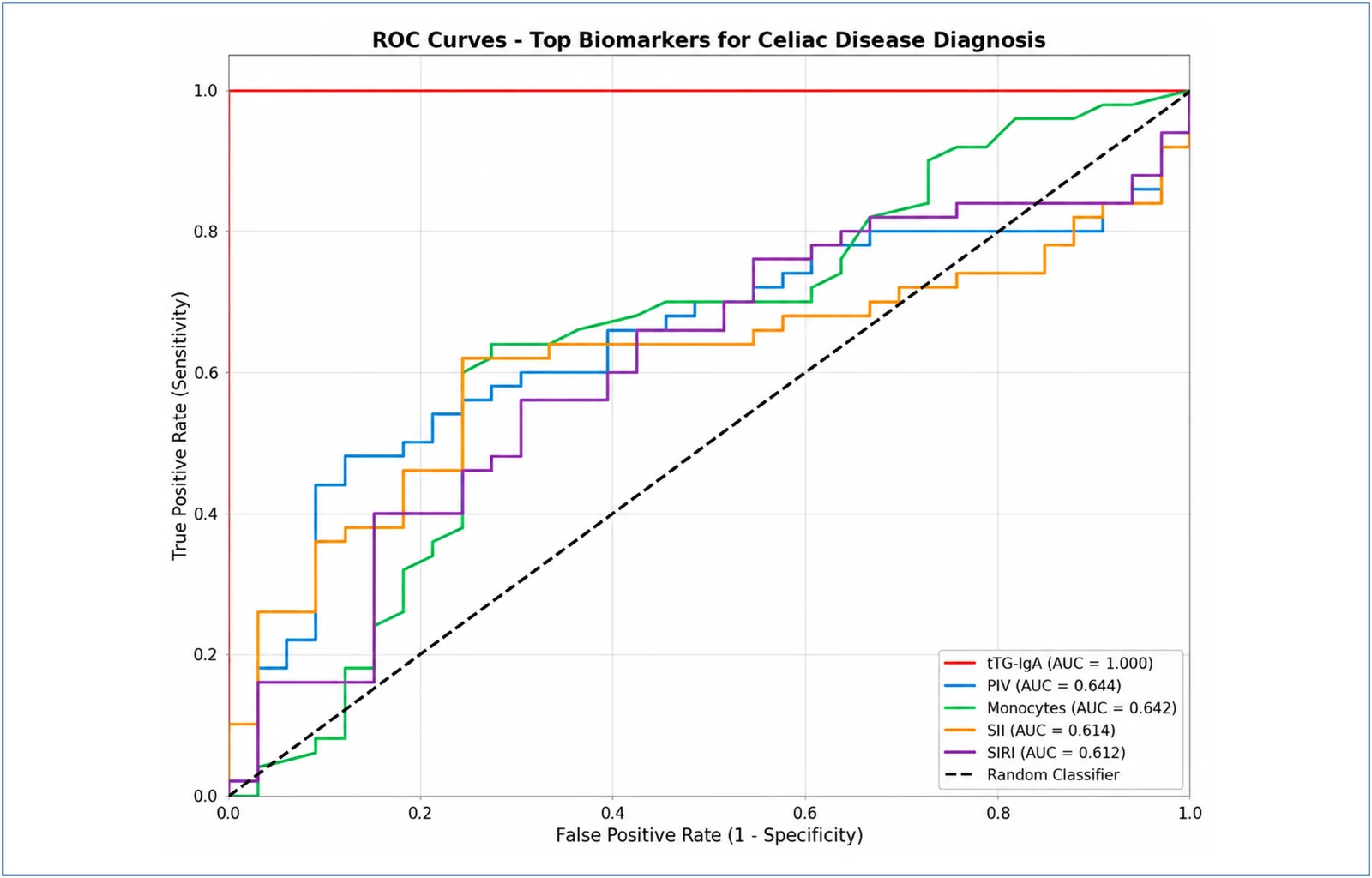
Receiver operating characteristic curves describing discrimination between the selected celiac and control groups. The tissue transglutaminase immunoglobulin A curve is shown for completeness; its AUC of 1.000 is determined by the study's inclusion criteria and must not be interpreted as diagnostic accuracy in an unselected population.

## DISCUSSION

In this cross-sectional pilot study, serum zonulin and three hemogram-derived inflammatory indices were higher in children with newly diagnosed celiac disease than in healthy controls. The findings support associations with altered intestinal permeability and systemic inflammation; they do not establish disease activity, longitudinal change, or independent diagnostic utility.

Zonulin regulates intestinal tight-junction permeability and has been investigated as a marker of barrier dysfunction in celiac disease. DaFonte et al. reported that zonulin concentrations increased before clinical diagnosis among children at genetic risk, supporting a possible relationship with early barrier alterations^6^. Martínez Gallego et al. evaluated fecal zonulin in pediatric celiac disease and reported changes during a gluten-free diet^12^. That study used a fecal matrix, whereas the present study measured serum zonulin. Fecal and serum measurements reflect different biological compartments and are affected by distinct pre-analytical and analytical factors; therefore, their concentrations and performance estimates should not be compared directly. Our finding of higher serum zonulin in the celiac group (p=0.024) is consistent with altered barrier function, but its cross-sectional association does not demonstrate mucosal severity or response to treatment.

Citrulline concentrations in the celiac group (31.76±9.85 μmol/L) were near commonly reported reference ranges and did not differ from controls. This contrasts with studies that have reported lower citrulline in newly diagnosed disease and inverse relationships with histological severity^10,14^. A plausible explanation is that the present pediatric cohort included predominantly mild or early mucosal involvement, with limited loss of enterocyte mass. This remains an inference because biopsy specimens were not collected and graded in a standardized manner. The absence of Marsh-Oberhuber staging prevents assessment of the relationship between citrulline and villous injury and precludes any claim regarding mucosal healing.

Hemogram-derived indices were higher in the celiac group, but their discriminatory performance was limited. Kıvrakoğlu et al. evaluated the SII in relation to histological stages of celiac disease^15^, whereas the present study could not stratify participants by biopsy severity. Differences in age, cohort composition, histopathological classification, and analytical thresholds may help explain why the AUCs in our pediatric sample (0.612–0.644) were lower than estimates reported in some prior studies. These AUCs indicate poor discrimination and should not be interpreted as evidence that the indices are clinically useful diagnostic tests. At most, they may provide adjunctive information about systemic inflammation when interpreted with established clinical and serological findings.

The strong correlations among the SII, SIRI, and PIV require particular caution. All three indices are algebraic combinations of neutrophil, lymphocyte, monocyte, and platelet counts; consequently, substantial correlation and multicollinearity are expected mathematically. The observed SIRI-PIV correlation (ρ=0.91) should therefore not be regarded as an independent clinical finding. Simultaneous clinical use of several highly correlated indices is unlikely to add information, and future studies should assess their incremental value in appropriately specified multivariable models before identifying the most parsimonious index for practice.

This study has several limitations. Most importantly, standardized duodenal histopathology and Marsh-Oberhuber classification were unavailable; therefore, biomarker concentrations could not be related to villous atrophy, mucosal severity, or mucosal healing. The cross-sectional design precludes assessment of disease activity over time or biomarker change after initiation of a gluten-free diet. Moreover, tTG-IgA positivity was an inclusion criterion for the celiac group and negativity was an inclusion criterion for controls. Its AUC of 1.000 is therefore a direct mathematical consequence of participant selection and does not represent its diagnostic accuracy in an unselected population. Analyses of the other markers should be interpreted within this selection framework. The modest sample size, single-center design, absence of a multivariable analysis of incremental biomarker contribution, and lack of standardized information on clinical phenotype further limit inference and generalizability. Accordingly, these findings should be viewed as proof-of-concept evidence that requires confirmation in prospective studies with biopsy-defined severity and longitudinal follow-up.

## CONCLUSION

Zonulin and hematological inflammation indices are potential markers associated with increased intestinal permeability and systemic inflammation in pediatric celiac disease. Together with tTG-IgA, these parameters may have an adjunctive role in reflecting disease-related pathophysiological processes; however, their low discriminatory performance precludes their use as stand-alone diagnostic tests.

## INFORMED CONSENT STATEMENT

Written informed consent was obtained from the parents or legal guardians of all pediatric participants before enrolment. The study protocol was conducted in accordance with the ethical principles of the Declaration of Helsinki.

## Data Availability

The datasets generated and/or analyzed during the current study are available from the corresponding author upon reasonable request.
